# Value of the New Spline QTc Formula in Adjusting for Pacing-Induced Changes in Heart Rate

**DOI:** 10.1155/2018/2052601

**Published:** 2018-04-01

**Authors:** Hirmand Nouraei, Matthew Bennett, Simon Rabkin

**Affiliations:** Department of Medicine (Cardiology), University of British Columbia, Vancouver, BC, Canada

## Abstract

**Aims:**

To determine whether a new QTc calculation based on a Spline fit model derived and validated from a large population remained stable in the same individual across a range of heart rates (HRs). Second, to determine whether this formula incorporating QRS duration can be of value in QT measurement, compared to direct measurement of the JT interval, during ventricular pacing.

**Methods:**

Individuals (*N*=30; 14 males) aged 51.9 ± 14.3 years were paced with decremental atrial followed by decremental ventricular pacing.

**Results:**

The new QTc changed minimally with shorter RR intervals, poorly fit even a linear relationship, and did not fit a second-order polynomial. In contrast, the Bazett formula (QTcBZT) showed a steep and marked increase in QTc with shorter RR intervals. For atrial pacing data, QTcBZT was fit best by a second-order polynomial and demonstrated a dramatic increase in QTc with progressively shorter RR intervals. For ventricular pacing, the new QTc minus QRS duration did not meaningfully change with HR in contrast to the HR dependency of QTcBZT and JT interval.

**Conclusion:**

The new QT correction formula is minimally impacted by HR acceleration induced by atrial or ventricular pacing. The Spline QTc minus QRS duration is an excellent method to estimate QTc in ventricular paced complexes.

## 1. Introduction

The accurate assessment of the QT interval is important to identify individuals at risk for potentially fatal arrhythmias in many conditions ranging from drug-induced cardiac toxicity [1–3] to genetic mutations of cardiac ion channels [4]. There are two major issues with respect to QT interval measurement, aside from problems with estimating the end of the T wave. First is adjusting for the heart rate dependency of the QT interval; despite utilization of one of the many previously available heart rate correction formulae, because each has significant limitations [5]. A second major problem is the lack of reliability of the QT interval measurement in the presence of ventricular pacing or bundle branch block.

Recently, a new QT-heart rate correction formula was developed based on the ECGs from about 13,627 individuals in the U. S. National Health and Nutrition Examination Survey (NHANES) population study and was shown to be relatively independent of heart rate and was superior to other formulae [6]. The limitation of large population-based databases is that they do not address intraindividual variability. The value of assessing individual responses to test the robustness of the QT-heart rate correction formulae has been recognized [7]. The new QT-heart rate adjustment formula requires assessment in persons using protocol driven changes in heart rate, such as cardiac pacing, in order to determine whether this QTc is stable when heart rate is increased [8].

Another important problem with QT interval measurement occurs in patients with ventricular pacing which makes it difficult for clinicians to determine whether the QT interval is prolonged. Calculating the JT interval has been proposed as a clinical method to overcome this problem [9]. Some investigators contend that in patients with ventricular pacemakers the JTc, and not the QTc, interval is the more useful marker for assessing repolarization [10]. However, population-based data in persons with ventricular conduction defects concluded that the JT adjustment assessed as QTc−QRS “retained a strong residual correlation with ventricular rate making its use ill-advised” [11].

The objective of this study was to determine whether QTc values calculated by the new QT correction formula remain relatively stable in the same individual across a range of heart rates in the setting of atrial pacing. The second objective was to determine how modification of this formula taking into consideration QRS duration would compare to direct measurement of the JT interval in conduction delay induced by ventricular pacing.

## 2. Methods

### 2.1. Patients and Pacing Protocols

Individuals (*N*=30; 14 males) with average age of 51.9 ± 14.3 years referred for cardiac electrophysiology study for the suspicion of supraventricular tachycardia with no evidence of ventricular preexcitation were included. All patients were brought to the electrophysiology laboratory at Vancouver General Hospital, Vancouver, BC, Canada, after having fasted for at least 8 hours. Patients were given sedation by constant intravenous infusion by an infusion pump so that no part of the pacing protocol would have been differentially affected by the sedation. Surface electrocardiogram recording was performed using ECG electrodes in the standard position with all patients in the supine position. Three right femoral and one left subclavian vein cannulations were performed. Catheters were inserted into the coronary sinus (CSL decapolar catheter, St. Jude Medical, St. Paul, MN, USA), His position (CRD-2 catheter, St. Jude Medical, St. Paul, MN, USA), and right ventricular apex (JSN, St. Jude Medical, St. Paul, MN, USA). The study was approved by the University of British Columbia Clinical Research Ethics Board. Patients had given consent for the procedure which was part of their investigation for the diagnosis of supraventricular tachycardia.

The ventricular pacing protocol was briefly as follows: decremental ventricular pacing beginning at a cycle length that was 100 ms faster than the sinus rate and continuing until VA block was achieved followed by ventricular extrastimulus pacing at 600 ms and 400 ms drive trains.

The atrial pacing protocol, which followed ventricular pacing, was briefly as follows: atrial pacing with atrial extrastimulus pacing at 600 ms and 400 ms drive trains. Decremental atrial pacing followed beginning at a cycle length that was 100 ms faster than the sinus rate and continuing until AV block was achieved. The pacing protocol was completed within 10 to 15 minutes.

### 2.2. Electrocardiogram Measurements

Electrocardiograms were recorded throughout the study using CardioLab®, version: 6.8.1, General Electric, Boston. For each patient, the RR and QT intervals were measured during atrial pacing and the RR, QRS, and QT intervals were measured during ventricular pacing. All intervals were measured on an ECG recorded at a paper speed of 100 mm/sec, using machine calipers. Two measurements, by a single individual (HN) to eliminate interobserver variability, were made for each interval, and the mean value was used. The QRS complex was measured as the interval between the initial QRS depolarization and the J point. The QT interval was measured beginning at the initial QRS depolarization and ending at the end of the T wave as outlined previously [12].

The QTc for atrial paced data was corrected using the new spline QTc (Spline), https://elenaszefer.shinyapps.io/qtc_nhanes_spline/ [6]. For comparison, the most commonly used QT correction formula was also calculated, namely, the one proposed by Bazett (QTcBZT) which is QT/(RR interval)^½^ [13]. A comparison QTc formula was used because the effect of the pacing protocol should equally affect any QT adjustment formula as the formulae are mathematical corrections of the underlying QT-RR relationship. The spline formula is a regression spline model predicting QT from heart rate and gender and was fit to the data using the least squares criterion. The linear model had seven orthogonal b-spline basis functions, *B*_1_(*x*),…, *B*_7_(*x*), taking heart rate as their argument and allowing for a nonlinear regression relationship to be fit between heart rate and QT. The analytic form of the b-spline functions is given in the supplement in the original paper [6].

The ventricular paced data were corrected using two separate methods. In the first method, the QTc was first determined by the new spline and Bazett formula and the JT interval was calculated by subtracting QRS duration from the corrected QT. In the second method, the JT interval was calculated by subtracting the QRS duration from the uncorrected QT. Next, the JT interval was corrected using the Spline or Bazett formulae.

### 2.3. Data Analysis

For each patient, the RR interval and the QT interval were measured at each pacing rate or RR interval. Two approaches were used. First, the average (mean and SD) was calculated for each RR pacing interval. In the second approach, the data for each individual were fit with a first or second-order polynomial. For both atrial and ventricular pacing, the corrected QT values were compiled and correlated with the measured RR interval. Linear and nonlinear least squares regression models were used to examine the QTc versus heart rate relationship (GraphPad Prizm version 7; GraphPad Prism Software, Inc., La Jolla, CA, USA). A first-order polynomial or linear approach was used first. A second-order polynomial was the main nonlinear model used because of the data, based on many different functional forms, that a second-order polynomial characterizes the QTc data and higher-order polynomial do not improve the fit [14]. The data are presented as mean ± 1 SD. To compare the individual patient data, the distribution of the slopes for the linear fit and the *B*_1_ and *B*_2_ parameters for the second-order polynomial were examined and compared using the Wilcoxon matched pairs nonparametric test.

## 3. Results

### 3.1. Comparison of the QTc Formulae for Atrial Paced Data

**Figure 1:**
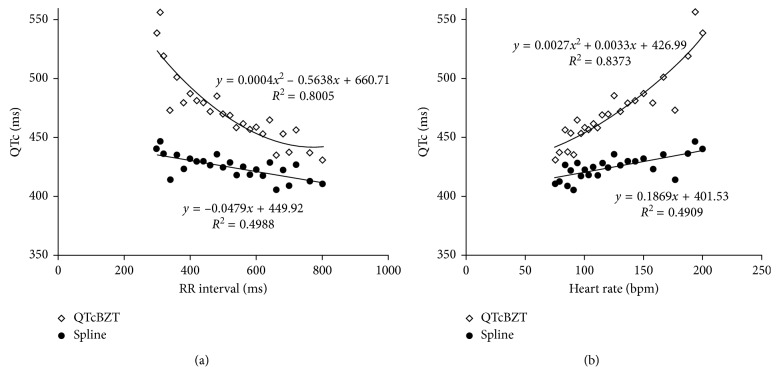
The relationship in atrial pacing between QTc for the Bazett versus new spline for pacing rate (RR) interval on (a) and expressed as heart rate on (b).

The relationship between QT interval and atrial pacing rate (RR interval) was analyzed comparing the QT correction based on the new spline formulae and on the Bazett formula (QTcBZT). The data are the mean of all patients at each pacing RR interval and are also presented as heart rate for ease of extrapolation (Figure 1). There was a weak linear relationship between the new spline QTc and RR interval (*R*^2^=0.4988) or heart rate (*R*^2^=0.4909). A second-order polynomial fit suggested an even weaker relationship between the new spline QTc and RR interval or increases in heart rate. These data suggest that the QTc spline formula is not strongly related to RR interval or heart rate. In contrast, shorter RR intervals were associated with progressively greater QTcBZT. QTcBZT increased progressively more (nonlinearly) with shorter RR intervals or increasing heart rate. The QTcBZT-RR relationship was best fitted with a second-order polynomial (*R*^2^=0.8005; *p*=0.0187) compared to a first-order polynomial or straight line (*R*^2^=0.742). The second-order polynomial fit for the relationship between the QTcBZT and heart rate also demonstrated a high correlation (*R*^2^=0.8373). In contrast, the new QT spline did not fit a second-order polynomial.

Comparison of the new QTc spline and QTcBZT needed to consider the different relationships to RR interval for each of the two different QTc.

**Figure 2:**
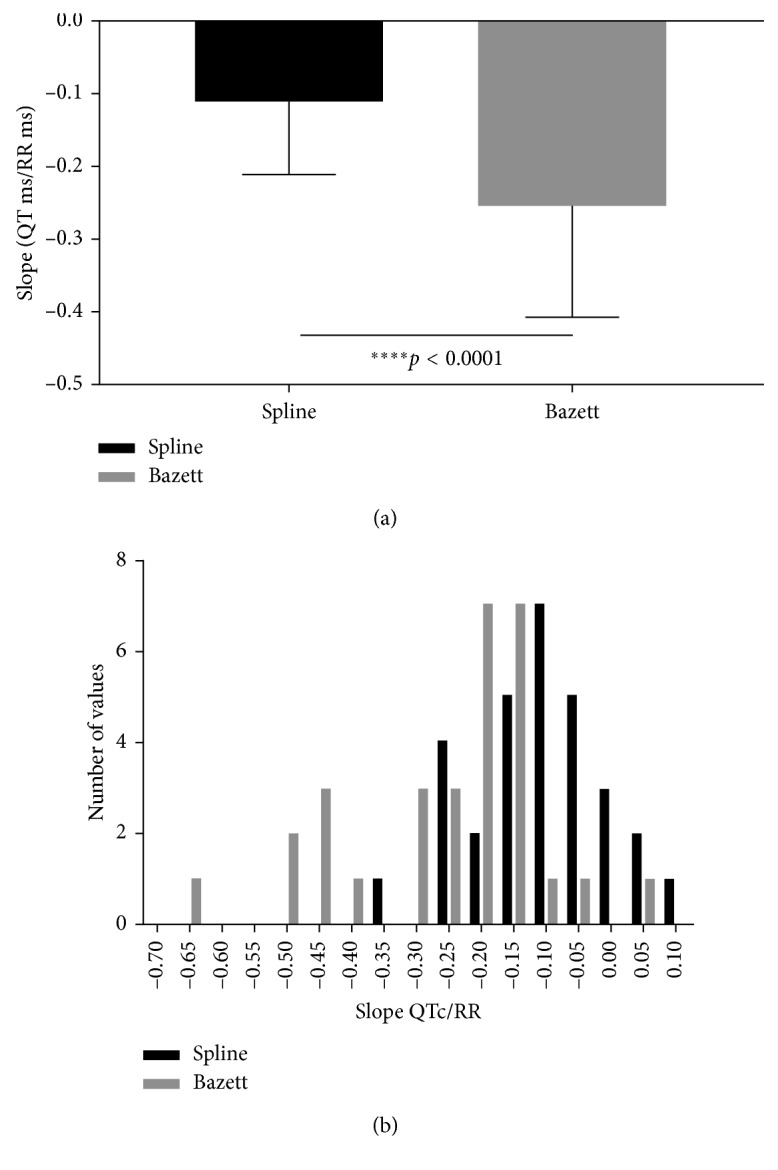
The slope of the relationship between QTc and RR interval for the new spline and Bazett formulae (mean and 1 SD) (a) and slope distribution (b).

In the next analysis, the data for each individual were evaluated. The relationship between the QTc Spline and QTcBZT with the RR interval was examined for each individual by fitting two different models: a linear or first-order polynomial and a second-order polynomial. The main parameter of the linear model was the slope, and the main parameters defining the second-order polynomial are *B*_1_ and *B*_2_. Each model was applied to both the QTc Spline and QTcBZT. Considering a linear model, the slope of the relationship between the spline QTc and heart rate was close to zero, that is, there was no relationship (Figure 2). In contrast, for QTcBZT there is a definite negative slope and one which was significantly greater or different from the Spline QTc. Examining the distribution of the slopes showed that a preponderance of values for the slope of the relationship were larger (more negative values) for QTcBZT compared to the new spline QTc. This is evidenced by the QTc/RR distribution which demonstrated higher values for QTcBZT (Figure 2(a)). Indeed there was a highly significant (*p* < 0.0001) difference between the two with the QTcBZT showing a much greater slope or stronger relationship between QTc and RR interval (Figure 2(b)). The negative slope reflects the greater QTcBZT at shorter RR intervals.

**Figure 3:**
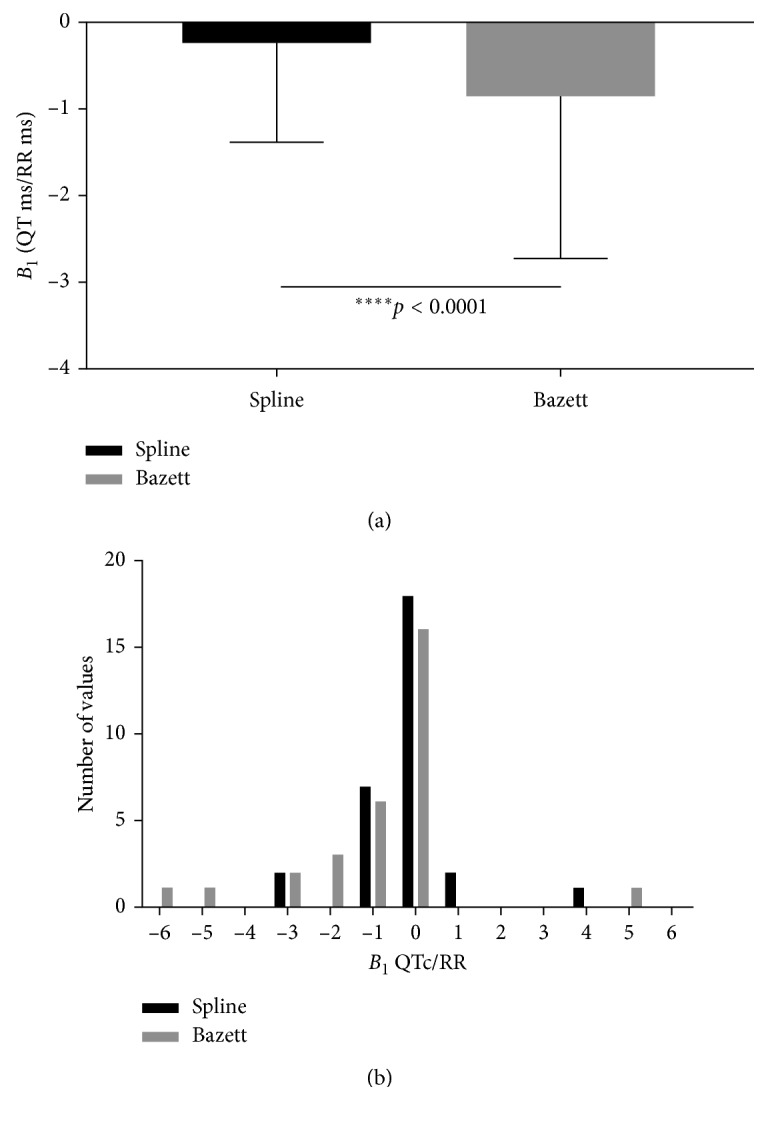
The *B*_1_ (mean and 1 SD) (a) and the distribution (b) for the second-order polynomial fit for atrial pacing.

**Figure 4:**
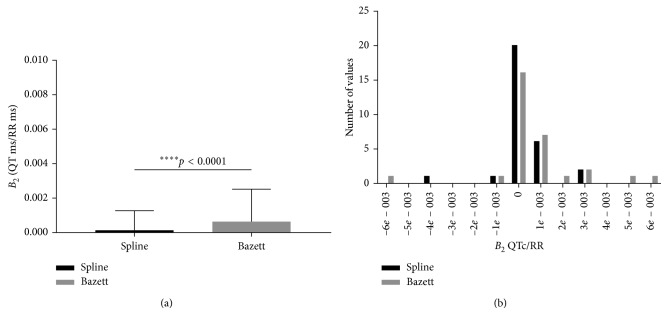
The *B*_2_ (mean and 1 SD) (a) and the distribution (b) for the second-order polynomial fit for atrial pacing.

We next considered both of the relationships as a second-order polynomial. The parameters *B*_1_ and *B*_2_ for the polynomial model demonstrated that these parameters for QTc Spline lie closer to zero, that is, a flat line (Figures 3 and 4). In contrast, these parameters were much larger for QTcBZT. Examination of the distribution of the slopes for the individual *B*_1_ and *B*_2_ for the polynomial model demonstrated that these parameters for QTc Spline lie closer to zero, that is, a flat line compared to QTcBZT. Restated, there were more individuals with zero slope parameters with the new spline QTc compared to QTcBZT which had greater numbers of individuals with larger values for these parameters (Figures 3 and 4). The second-order polynomial (*y* = *B*_0_ + *B*_1_*x* + *B*_2_*x*^2^), displayed a significantly greater *B*_1_ (*p*=0.0002) and *B*_2_ (*p*=0.0022) for QTcBZT compared to the new spline QTc demonstrating that QTcBZT had a stronger relationship to the pacing RR interval (Figures 3 and 4).

Thus, the two different approaches presenting the mean of the QTc at each RR interval (Figure 1) and the individual data on the QT–RR (heart rate) relationship (Figures 2–4) provide consistent evidence that the new Spline method is not impacted much by different pacing rates or heart rates especially when compared to QTcBZT.

### 3.2. Comparison of the QTc Formulae for Ventricular Paced Data

**Figure 5:**
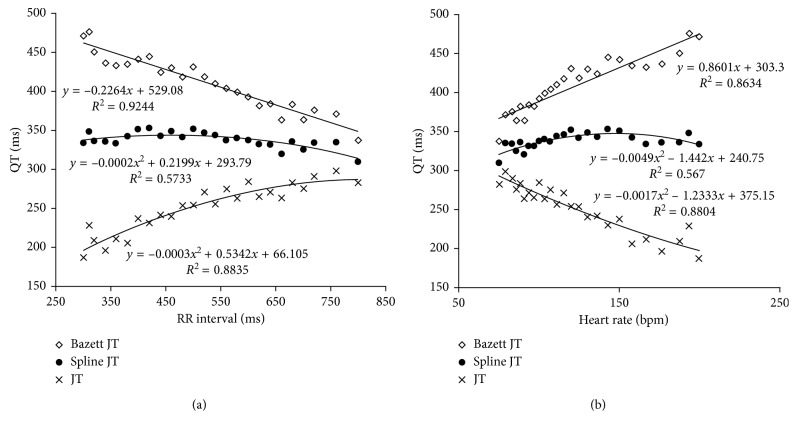
The relationship during ventricular pacing between the QTc for the Bazett versus new spline formulae for pacing rate (RR) interval on (a) and expressed as heart rate on (b). The data are shown for the Bazett JT, Spline JT (derived by calculating the QTc and subtracting the measured QRS duration), and the measured JT.

To analyze the QT interval in ventricular pacing, the data were analyzed using the same approach as in Figure 1 and two different analytic techniques were used. In the first approach, the ventricular paced QT data were corrected by the Bazett or new spline formulae and the QRS duration was subtracted from the calculated value—the adjusted approach (Figure 5). The JT data were calculated as unadjusted QT minus QRS and is shown for comparison. The adjusted approach using the corrected QT with the Bazett formula and subtracted from QRS was best fitted using a straight line (*R*^2^=0.9244; *p* < 0.0001) with a negative slope. QTc ranged from 337 to 471 ms. There was a marked increase in QTc with shorter RR intervals or faster heart rates. In contrast, the adjusted approach using the QTc calculated by the new spline formula minus the QRS remained relatively stable with shorter RR intervals or faster heart rates with a narrow range, 309 to 333 ms. The QTc spline subtracted from QRS was best fitted with a second-order polynomial (*R*^2^=0.5733; *p*=0.0018), but the relationship was weaker than the QTcBZT as it was relatively flat. The JT data, which was calculated as unadjusted QT minus QRS, showed a positive slope and a large range, from 187 to 282, across the pacing range. Thus, the JT was in the opposite direction of the QTcBZT but showed the similar large range of 100 ms, which was slightly less compared to QTcBZT of 130 ms. In contrast, the new spline correction showed the smallest, 24 ms, difference from the highest to the lowest RR interval.

**Figure 6:**
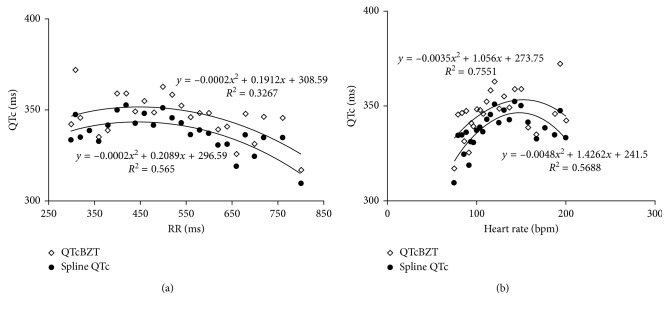
The relationship during ventricular pacing between QTc for the Bazett versus new spline for pacing rate (RR) interval on (a) and as heart rate on (b). The QT minus QRS was entered into the calculation for the QTc formula for the Bazett JT and Spline JT. The data are expressed at QTc-QRS.

The second approach for evaluation of the ventricular paced QT data was to first subtract the QT from QRS and then use the QTcBZT or the new spline QT formula (Figure 6). In this method, the QT interval with ventricular pacing showed a curvilinear relationship. Both sets of data displayed a heart rate dependency characterized by a pattern of increases in the corrected QT with faster heart rates followed by a decrease in QTc with progressively faster heart rates or shorter RR intervals.

## 4. Discussion

This study presents important data on which to change the standard way the QT interval is adjusted for heart rate in cardiology practice. It is the first to test the new spline QTc formula in the setting of programmed increases in heart rate using atrial and ventricular pacing. In so doing, it has shown that the spline QT formula is relatively resistant to the impact of changes in heart rate. We used atrial pacing to achieve changes in heart rate. In addition, we developed a new approach to the estimation of the QTc in the presence of conduction delay induced by ventricular pacing. The new spline QTc minus the QRS duration was relatively insensitive to the influences of changes in heart rate and should be useful for estimation of QT interval in ventricular paced rhythm and perhaps as well in QRS prolongation from bundle branch block.

The new formula is based on functionally agnostic modeling of a population QT–heart rate relationship using flexible regression splines. Previous formulae used standard mathematical functional forms which “force the data” to fit a known relationship which likely explains why those QTc formulae do not accurately adjust for the effect of a wide range of heart rates. In contrast, the new formula does not presuppose a specific form (quadratic, logarithmic, and so on) but rather used the data to construct a new formula which eliminated the impact of heart rate on QT interval and was better than other formulae [6].

The QT interval on the surface ECG is a composite of ventricular activation (QRS) and completion of repolarization (end of the T wave) as well as the net effect of these forces reaching the body surface [15]. While the QT interval may not be a direct expression of a single-cardiac physiologic property [15], inhibition of cardiomyocyte inward currents by a number of drugs produces QT prolongation and the potentially fatal arrhythmia of Torsade de Pointes [16]. The association of drug-induced QT prolongation and potentially fatal arrhythmias [17] highlights the need for accurate QT interval measurement. If a QT-heart rate correction formula does not accurately correct for the impact of heart rate on QT interval, it will lead to an over- or underestimate of the impact of a drug on cardiac repolarization and its potential for induction of cardiac arrhythmias.

The new spline formula for correction of QT demonstrates a more reliable and less variable outcome compared to the Bazett formula. We chose the Bazett formula as a comparator because it is the formula that is in most wide spread usage. Computerized QTc correction in many computer systems provides only the Bazett formula. If the new spline QTc is to be translated into clinical practice, it must be compared to the QTc formula that is most generally used. The ultimate goal of the QT correction formula is to make the QT interval independent of heart rate. To that extent, in atrial pacing, the new spline QTc showed a linear relationship with heart rate, but it was only weakly associated with heart rate suggesting that it is close to being independent of heart rate. In atrial pacing, the new spline QTc, when fit with a linear relationship, had minimal range and variation. In other words, the new spline QTc showed a small range from the maximum to the minimum heart rate equivalent to a minimal impact of heart rate on the QT interval. While the data were fit with a straight line, the relationship was not strong. In contrast, QTcBZT was well fit to a polynomial relationship. Shorter RR intervals were associated with progressively greater QTcBZT. The new spline method minimizes variation of QTc with RR interval and reduces variability at the extremes of the RR interval. In contrast, QTcBZT magnifies the increase in QTc with faster heart rates.

For ventricular pacing, if the QT is corrected first and then subtracted from QRS duration, the result is that new spline QTc data are in a narrow range with less variability compared to QTcBZT. In contrast, if the JT is calculated first and is inserted into the formulae, both formulae are not useful as they generate corrected values that are fitted with a second-order polynomial and both displayed a pattern of increase in the corrected QT with the RR interval followed by a decrease in the corrected QT. Subtraction of the QRS from the unadjusted QT and entering the result into the correction formulae, as anticipated, is not a good approach because both the new QTc spline and the QTcBZT were developed on the basis of having a measurement from the onset of the QRS.

Several approaches have been proposed to correct the QT interval in persons with wide QRS complexes as exemplified by ventricular pacing. Chakravarty et al. examined the suggestion of subtracting 50 ms from the QTc interval and reported that this approach was reasonable for cases with heart rates of 66 bpm but not at faster heart rates [18]. Although subtracting 50 ms has the advantage of simplicity, the 50 ms adjustment underestimates QTc [18]. Some studies suggest that the corrected JT (JTc) interval may be a more accurate measure of ventricular repolarization in the setting of a prolonged QRS [19–21]. We found that JTc was not a good approach because it demonstrated heart rate dependency. In ventricular pacing, we found that the JT interval shortened, while QT prolongs consistent with the data of Chiladakis et al. [22]. Taken together, our data support a new approach to first calculate QTc using the new spline QTc formula and then subtract the QRS duration, rather than using JT measurement for assessment of repolarization in ventricular pacing.

Our data suggest that QTcBZT is not a valid approach for QT correction across the range of heart rates. This finding is consistent with others who concluded that the Bazett correction formulae is inaccurate leading to “over correction at high rates and under correction at low rates” [23] and suggest that Bazett's formula should not be applied for the correction of QT changes induced by atrial or ventricular pacing [23, 24]. We extend these observation and as well present data that the new spline QTc overcomes the problems of QTcBZT.

The data on the value of the spline QT raise questions for future research. Studies on the potential value of the new QTc formula to identify the role of heart rate in predicting or precipitating malignant arrhythmias should be studied. Clinical studies of patients with long QT syndromes should also evaluate the new QTc formulae. Epidemiologic studies of large populations should evaluate the new QT spline formula as a predictor of sudden cardiac death and to determine whether it is a more precise predictor of sudden death than QTcBZT.

### 4.1. Limitations of the Study

There are several limitations of the study that warrant discussion. While pacing produces a controlled increase in heart rate, it is a relatively short-term change and the effects on the QT interval may be different under conditions of changes in heart rate of longer durations. This issue is especially relevant to the issue of QT/RR hysteresis which is the lag of adaptation of the QT interval to heart rate changes. There is unfortunately no consensus on the optimal method for quantification of QT/RR hysteresis as recently outlined by Gravel et al., and there is a pressing need for a rigorous application-specific comparison of currently proposed methods [25]. For this reason and for the possibility that the nature of the pacing protocol may have influenced the resulting QT/RR correction, our study compared two different QT correction formulae as they should be equally affected by the impact of QT/RR hysteresis. Our finding of a marked discrepancy between the Spline formula and QTcBZT supports the contention that the Spline approach is the preferred method for calculation of QTc. Second, ventricular pacing was undertaken using pacing catheter placed at the right ventricular apex. Whether the findings might be different with other kinds of ventricular pacing sites is unknown. Pacemaker leads, however, are usually placed in the right ventricular apex, so the data should be relevant for most pacemaker situations. Third, the method for inducing heart rate changes utilized cardiac pacing. While this is an artificial method for heart rate changes, it has the advantage of achieving changes in heart that are relatively independent of changes in autonomic tone or catecholamines which may occur in other situations such as in exercise-induced changes in heart rate [26]. The ultimate test of the new QTc will be further research which demonstrates its ability to improve the prediction of fatal cardiac arrhythmias after considering other predictors of fatal arrhythmias such as left ventricular ejection fraction. Lastly, it should be emphasized that the value of the new corrected J-T approach which first corrects the QT interval and then subtracts the QRS duration was validated in a specific pacing mode with pacing in the apex of the right ventricle. We speculate but do not have data that this QT adjustment process will also be useful to provide the kind of data to calculate the QT interval in situations with pacing catheters in other locations or in bundle branch block.

## 5. Conclusion

There are several major conclusions from this work. First, the new QT correction formula is minimally impacted by increases in heart rate induced by atrial and ventricular pacing. The new formula is a reliable correction method for the QT interval and is suitable for minimizing the impact of heart rate on QTc. It should supplant QTcBZT in clinical practice. Second, the QTcBZT is a poor approach to correct for the effect of shorter RR intervals or faster heart rates on the QT interval. Third and importantly, the new QTc minus QRS duration is an excellent approach to estimate QTc in the presence of wide QRS complexes from ventricular pacing. We suggest that the approach might be translated to other conditions with prolonged QRS duration and suggest that this is an area for further investigation.
